# Telephone counselling by nurses in Norwegian primary care out-of-hours services: a cross-sectional study

**DOI:** 10.1186/s12875-017-0651-z

**Published:** 2017-09-06

**Authors:** Vivian Midtbø, Guttorm Raknes, Steinar Hunskaar

**Affiliations:** 1 National Centre for Emergency Primary Health Care, Uni Research Health, Box 7810, NO 5020 Bergen, Norway; 2 Regional Medicines Information & Pharmacovigilance Centre (RELIS), University Hospital of North Norway, Box 79, NO 9038 Tromsø, Norway; 3 Raknes Research, Myrdalskogen 243, NO 5117 Ulset, Norway; 4 Department of Global Public Health and Primary Care, University of Bergen, Box 7804, NO 5018 Bergen, Norway

**Keywords:** Telephone counselling, Primary health care, After-hours care, Nurse, Reason for encounter, International classification of primary care, Norway

## Abstract

**Background:**

The primary care out-of-hours (OOH) services in Norway are characterized by high contact rates by telephone. The telephone contacts are handled by local emergency medical communication centres (LEMCs), mainly staffed by registered nurses. When assessment by a medical doctor is not required, the nurse often handles the contact solely by nurse telephone counselling. Little is known about this group of contacts. Thus, the aim of this study was to investigate characteristics of encounters with the OOH services that are handled solely by nurse telephone counselling.

**Methods:**

Nurses recorded ICPC-2 reason for encounter (RFE) codes and patient characteristics of all patients who contacted six primary care OOH services in Norway during 2014. Descriptive statistics and frequency analyses were applied.

**Results:**

Of all telephone contacts (*n* = 61,441), 23% were handled solely by nurse counselling. Fever was the RFE most frequently handled (7.3% of all nurse advice), followed by abdominal pain, cough, ear pain and general symptoms. Among the youngest patients, 32% of the total telephone contacts were resolved by nurse advice compared with 17% in the oldest age group. At night, 31% of the total telephone contacts were resolved solely by nurse advice compared with 21% during the day shift and 23% in the evening. The share of nurse advice was higher on weekdays compared to weekends (mean share 25% versus 20% respectively).

**Conclusion:**

This study shows that nurses make a significant contribution to patient management in the Norwegian OOH services. The findings indicate which conditions nurses should be able to handle by telephone, which has implications for training and routines in the LEMCs. There is the potential for more nurse involvement in several of the RFEs with a currently low share of nurse counselling.

**Electronic supplementary material:**

The online version of this article (doi:10.1186/s12875-017-0651-z) contains supplementary material, which is available to authorized users.

## Background

The primary care out-of-hours (OOH) services in Norway are characterized by high contact rates by telephone [1, 2]. Telephone contacts are handled by local emergency medical communication centres (LEMCs). The LEMCs are most commonly staffed by registered nurses, who triage the contacts and refer the patients to the appropriate level of care. If the patient’s condition does not require assessment by an on-call medical doctor, the nurse often handles the contact solely by telephone counselling. In most of the 200 or so Norwegian primary care OOH districts, the only way to contact the OOH services is by first calling the LEMC. In several of the largest cities patients can contact OOH casualty clinics at all times (24/7), and may turn up directly without calling the LEMC in advance. Direct attendance is, however, not encouraged. The OOH system is organized within the primary health care sector, owned by the municipalities, and serves as a gatekeeper to the hospitals and other secondary health care, which are owned by the state. Medical responsibility for the services is mainly assumed by general practitioners (GPs) who take shifts in their own OOH district, and by interns serving their compulsory general practice period.

Because of the challenging geography in parts of Norway, with poor roads, islands without road connection to the mainland, and scarcely-populated rural areas, the size of the OOH organisations varies considerably. The smallest organisation serves a population of less than a thousand people, while the largest casualty clinic in the capital, Oslo, serves more than 600,000.

The nurses’ contribution to the LEMCs constitutes an important part of the primary care OOH services. It is estimated that there were approximately 1.8 million contacts with the OOH services in Norway in 2013 (contact rate 0.36 per inhabitant per year) and about 75% of these contacts were telephone contacts to an LEMC (0.27 contacts per inhabitant per year) [2]. Based on estimates, less than a third of these contacts were handled solely by nurse telephone counselling, with a wide variation between the LEMCs [2]. Nevertheless, this is an important and large volume activity that educates and counsels patients and caregivers, and reduces casualty clinic workload.

Previous literature on nurses’ triage and telephone counselling in the OOH services has focused on the quality of communication and decisions [3–7], the safety of telephone triage [8–10], the use of protocols and decision support tools [11–16], the process of giving advice over the telephone versus meeting the patient face-to-face [17], patients’ satisfaction with the advice, and their understanding and adherence to the advice [18–21]. Overall, previous research has found nurse telephone triage to be safe [8, 21]. Nonetheless, the nature of telephone triage makes it a vulnerable part of the service because the patient is not seen face-to-face. The level of nurse telephone advice differs between countries, ranging from about 23% in Norway [2, 22] to 40–50% in the Netherlands and UK [18, 23]. Studies have found that the quality of the nurses’ advice is satisfactory [7], and that the patients understand and follow the advice given by the nurses [19–21, 24].

Because the organisation of primary care services varies, it can be difficult to directly compare nurse telephone triage between countries. Many of the European countries have a strong primary care health service, where triage nurses cover the whole range of contact types. In countries where the primary care service is less built out, such as in the United States, additional nurse triage services are often run from the specialized health care services, aimed at specific patient groups, such as paediatrics [24–26]. However, some findings are similar across continents. Studies from Europe, the United States, New Zealand and Australia all show that children constitute a large group of the OOH contacts across the different continents [2, 24, 27–29]. Frequent RFEs in this group include fever, vomiting, colds and cough [24, 27, 28].

There is a lack of epidemiological studies documenting which medical problems and reason for encounter (RFE) are resolved and handled by nurses’ telephone advice in the OOH services. Some European studies include information on the RFE using the ICPC-2 classification system [29–32]. In a Dutch study, researchers explored nurse telephone triage using the determinants of independent advice and return consultation [33]. Other than this, we have not found studies that investigate the characteristics of telephone contacts that were resolved by nurse telephone counselling.

Therefore, the present study aimed to identify which RFEs are most frequently handled by nurse telephone counselling in the OOH services. More specifically, we wanted to investigate which diagnostic chapters and single codes within the ICPC-2 are most frequently resolved by nurse telephone advice and, in addition, to examine a number of patient characteristics and the time of the encounter.

## Methods

A cross-sectional design was applied to this study. Patient data for one year (2014) were collected from six OOH districts to estimate the incidence of nurse counselling at different RFEs.

### Setting

The data were collected within a sentinel network called “The Watchtowers”, which was established in 2006 by the National Centre for Emergency Primary Health Care [34]. The network includes the LEMCs in a representative sample of seven Norwegian OOH districts, consisting of 18 municipalities. Information on all contact, both by telephone and direct attendance, to the participating centres is recorded in an online database (Zoho Creator®) by the nurses on duty at that time. For the purposes of this study we included six of the seven Watchtowers. One was excluded due to major organisational changes during the time of data collection. Approximately 150 nurses worked in the LEMCs of the six Watchtowers during the period of data collection. Most nurses working in the LEMCs were registered nurses, and had prior clinical work experience before starting work in the LEMC. All nurses in the study were involved in both telephone nurse counselling and clinical patient management in the OOH services where they worked. This means that, in addition to operating the telephone in the LEMC, they also had duties in regard to taking care of patients in the casualty clinic, such as triage, performing clinical observations, assisting the GP and laboratory work (such as blood samples and measuring C-reactive protein). The nurses rotated between operating the telephone and undertaking clinical work in the casualty clinic.

### Participants and study size

Study size was determined by the number of dispensed patient contacts handled by nurses in the Watchtower project in 2014. Because the study focusses on contacts handled solely by nurse telephone advice, only telephone contacts were included in the study. Contacts of direct attendance at the OOH service, or with unknown mode of contact, were excluded from the study.

### Variables

RFE was registered using the ICPC-2 (International Classification of Primary Care, 2nd edition). The ICPC-2 allows for classification of the patient’s RFE, problems, diagnoses and interventions. The ICPC system was developed by WONCA [35]. It was published in 1987 and has been used in Norway from 1992. In the Watchtower project, RFE is recorded based on the patient’s complaint or stated reason for contacting the OOH service. In order to minimize systematic variation, nurses at all participating casualty clinics have received standardized instructions on how to register ICPC-2 RFE codes in accordance with the official manual (http://www.kith.no/sokeverktoy/icpc-2/bok/kontaktarsak.html).

“Action taken” was classified in one of the following categories: (A) Telephone consultation/advice with a nurse or other health personnel, not a doctor, (B) Telephone consultation with a doctor, (C) Medical examination by a doctor, (D) Consultation with other than a doctor, (E) Call-out with a doctor and ambulance, and (F) Home visit by GP. The main focus of this study is on the contacts registered in the category: “Telephone consultation/advice with a nurse or other health personnel, not a doctor”. For the purposes of analysis, we refer to this as “contacts handled solely by nurse telephone counselling/advice”.

Mode of contact was classified in one of the following categories: (A) Telephone contact, (B) Direct attendance at the casualty clinic, (C) Contact with health professionals, (D) Contact with national emergency medical communication centres, or (E) Other.

Time of day was registered by three time categories: Daytime 08.00–15.29, afternoon 15.30–22.59, and night 23.00–07.59. We also recorded day of the week (Monday-Sunday), and the patient’s gender and age (under one year of age was recorded as “0”). The urgency level was assessed by the nurse in accordance with the Norwegian Index for Medical Emergency Assistance (Index) [36] into one of three urgency levels defined by colour: Red (acute), yellow (urgent) and green (non-urgent). Because of the low numbers of yellow (*n* = 892) and red (*n* = 19) urgency level cases handled by nurse telephone counselling we combined all levels of urgency in the analysis.

Because many of the ICPC-2 codes involve similar symptoms, we grouped some related ICPC-2 codes (see Additional file 1).

### Data analysis

All patient contact data from 2014 were downloaded from the online database (Zoho Creator®) in January 2015. Excel was used to organize the data before importing the files into IBM SPSS statistics 22. Descriptive statistics and frequency analyses were used. In addition, logistic regression analysis was used to explore the relationship between nurse telephone counselling and time of day, and age of the patients. Nurse telephone counselling (yes/no) was the dependent variable; time of day and age group were independent, categorical variables. Data are presented as frequencies, rate per 100,000 inhabitants/year and proportion of total telephone contacts.

### Ethics

The Watchtower project has been approved by the Regional Committee for Medical and Health Research Ethics, the Norwegian Social Science Data Services, and by the privacy ombudsman for research for Uni Research. The Watchtower database is owned and managed by our institution, the National Centre for Emergency Primary Health Care. No patient identifiable data were recorded at any time. The database was only accessible to the researchers in the project.

## Results

### Participants and contact characteristics

The six OOH districts included in the study had a combined population of 218,205 inhabitants as of 1 January 2014. During the study period, there were a total of 77,863 medical encounters, equivalent to 357 contacts per 1000 inhabitants per year. Of these, 16,394 attended the casualty clinic directly without contacting the LEMC beforehand, and 28 records had unknown mode of contact. Because the scope of this study is telephone contacts, direct attendance and unknown mode of contact were not included in the analysis. This gave a total of 61,441 encounters, equivalent to 282 telephone contacts per 1000 inhabitants per year, to be included in the analysis. These accounted for 79% of the total medical contacts.

14,155 (23%) of the telephone contacts were handled solely by nurse telephone counselling (65 contacts per 1000 inhabitants per year). The remaining telephone contacts were handled as follows: 10.2% had a telephone consultation with a doctor (29 contacts per 1000 inhabitants per year), 58.3% had a medical examination by a doctor (164 contacts per 1000 inhabitants per year), 1.2% had a consultation with other than a doctor (3 contacts per 1000 inhabitants per year), 2.6% had a call-out with a doctor and an ambulance (7 contacts per 1000 inhabitants per year), 1.4% had a home visit by GP (4 contacts per 1000 inhabitants per year), and 3% were other actions.

The age of the patients in the group that received nurse advice ranged from 0 to 103 years (median age of 25 years, mean age of 30.7 years), and women constituted 57.7% of the records. 1702 cases (12%) had no registered ICPC-2 code, and in 601 (4.2%) cases age was not registered.

### Telephone contacts handled by nurse counselling in the different ICPC-2 chapters

Table 1 presents the distribution of telephone contacts handled by nurse telephone counselling in each ICPC-2 chapter. Encounters related to chapter A (General and unspecified problems) were most frequently resolved by nurse telephone advice, followed by L (Musculoskeletal), D (Digestive), S (Skin) and R (Respiratory problems).

**Table 1:** Telephone contacts handled by nurse counselling within each ICPC-2 chapter

ICPC-2 chapter		Nurse telephone advice (*n* = 14,155)			Proportion	
		Frequency	(%)	Incidence	%	(95% CIs)
A	General and unspecified	2683	(19.9)	1230	27	(26–28)
L	Musculoskeletal	1788	(12.6)	819	21	(20–21)
D	Digestive	1681	(11.9)	770	27	(26–28)
S	Skin	1444	(10.2)	662	25	(24–26)
R	Respiratory	1392	(9.8)	638	18	(17–19)
P	Psychological	710	(5.0)	325	28	(26–30)
H	Ear	568	(4.0)	260	35	(32–37)
N	Neurological	543	(3.8)	249	21	(19–22)
U	Urological	448	(3.2)	205	13	(12–14)
F	Eye	437	(3.1)	200	19	(17–20)
W	Pregnancy and family planning	185	(1.3)	85	28	(24–31)
X	Female genital	168	(1.2)	77	31	(27–35)
K	Cardiovascular	160	(1.1)	73	10	(8–11)
T	Endocrine/metabolic/nutrition	77	(0.5)	35	22	(17–26)
Y	Male genital	63	(0.4)	29	19	(15–23)
Z	Social problems	59	(0.4)	27	23	(18–28)
B	Blood	47	(0.3)	22	27	(20–34)

The numbers are presented as frequency, incidence per 100,000 inhabitants/year and the proportion of total telephone contacts handled by nurse counselling per chapter

In chapters H (Ear) and X (Female genital), more than 30% of the total telephone contacts were resolved by nurse advice. In contrast, in chapters U (Urological) and K (Cardiovascular) less than 15% of the total telephone contacts were resolved by nurse advice.

The distribution of the ICPC-2 chapters most frequently handled by nurse telephone counselling was quite similar for day and evening shifts, with some small differences for the night shifts. Chapter A (General and unspecified) was the chapter most frequently resolved by nurse telephone advice during all three shifts. Chapters D (Digestive) and P (Psychological) constituted more of the contacts resolved by nurse telephone advice during night shifts compared to day and evening shifts.

### RFE ICPC-2 codes most frequently handled by nurse telephone counselling

For telephone contacts handled solely by nurse telephone counselling, 447 different ICPC-2 codes were recorded.

Table 2 presents the 15 RFE ICPC-2 codes most often handled solely by nurse telephone counselling. A03 (Fever) was most prevalent, accounting for 7% of all nurse advice in this study, and constituted 39% of all nurse advice given in chapter A. This was followed by D01 (Abdominal pain), R05 (Cough), H01 (Ear pain) and A29 (General symptoms). In eight of the RFE codes in Table 2 more than 30% of the total telephone contacts were resolved by nurse telephone advice. S18 (Laceration/cut) had the lowest total share in this group, with 11% of the total telephone contacts resolved solely by a nurse.

**Table 2:** The 15 RFE ICPC-2 codes most frequently handled by nurse telephone counselling

ICPC-2 code		Nurse telephone advice (*n* = 14,155)			Proportion
		Frequency	(%)	Incidence	%
A03	Fever	1039	(7.3)	476	32
D01	Abdominal pain	471	(3.3)	216	18
R05	Cough	431	(3.0)	198	22
H01	Ear pain	311	(2.2)	143	37
A29	General symptom	295	(2.1)	135	31
R21	Throat symptom	266	(1.9)	122	17
L17	Foot/toe symptom	240	(1.7)	110	23
D10	Vomiting	235	(1.7)	108	45
L02	Back symptom	228	(1.6)	104	23
N01	Headache	222	(1.6)	102	23
A13	Concern/fear of medical treatment	217	(1.5)	99	33
A97	Administrative contact	204	(1.4)	93	35
S18	Laceration/cut	197	(1.4)	90	11
D11	Diarrhoea	193	(1.4)	88	46
S06	Rash localized	187	(1.3)	86	37
	Other	7717	(54.5)	3536	21
	ICPC-2 unknown	1702	(12.0)	780	28
	All nurse telephone advice	14,155	(100)	6487	23

The numbers are presented as frequency, incidence per 100,000 inhabitants/year and the proportion of total telephone contacts handled by nurse counselling per code

Table 3 presents the 30 RFE groups and non-grouped RFE codes most frequently handled by nurse telephone counselling. The RFEs in Table 3, General symptoms, were most frequently resolved by nurse advice, followed by the non-grouped RFE code A03 (Fever) and the groups Lower limbs symptom/injury/condition, Respiratory symptom/condition, Abdominal pain and Fears/concerns and worries. Table 3 includes 80% of all nurse advice given with a known ICPC-2 code (71% of all advice given when contacts with unknown ICPC-2 code are included). Furthermore, it includes 168 (38%) of the 447 ICPC-2 codes in the category of telephone contacts handled by nurse telephone counselling.

**Table 3:** RFE groups and non-grouped ICPC-2 codes most frequently handled by nurse telephone counselling

RFE group/non-grouped ICPC-2 RFE code	Nurse telephone advice (*n* = 14,155)			Proportion
	Frequency	(%)	Incidence	%
General symptom	1053	(7.4)	483	23
Fever	1039	(7.3)	476	32
Lower limbs symptom/injury/condition	656	(4.6)	301	20
Respiratory symptom/condition	621	(4.4)	285	16
Abdominal pain	579	(4.1)	265	17
Fears, concerns and worries	567	(4.0)	260	33
Diarrhoea/Vomiting	499	(3.5)	229	44
Upper limb symptom/injury/condition	472	(3.3)	216	18
Ear symptom/condition	413	(2.9)	189	37
Neck/back symptom/condition	394	(2.8)	181	24
Skin itching/rash	389	(2.7)	178	35
Urinary tract symptom/condition	299	(2.1)	137	13
Skin injury	298	(2.1)	137	14
Throat symptom/condition	279	(2.0)	128	17
Eye symptom/condition	262	(1.9)	120	17
Head/face symptom/condition	243	(1.7)	111	22
Anxiety	231	(1.6)	106	33
Administrative contact	204	(1.4)	93	35
Mouth/teeth symptom/condition	177	(1.3)	81	41
Animal/human bite	168	(1.2)	77	37
Depression	150	(1.1)	69	25
Limited function/disability	143	(1.0)	66	34
Insect bite/sting	132	(0.9)	60	37
Respiratory infections	116	(0.8)	53	19
Constipation	116	(0.8)	53	49
Nose/sinus symptom/condition	106	(0.7)	49	24
Vertigo/dizziness	106	(0.7)	49	22
Alcohol/substance abuse/addiction	104	(0.7)	48	23
Chest symptom/condition	102	(0.7)	47	8
Allergy/allergic reactions	79	(0.6)	36	19
Other	2456	(17.4)	1126	21
ICPC-2 unknown	1702	(12.0)	780	28
All	14,155	(100)	6487	23

The 30 RFE groups and non-grouped ICPC-2 RFE codes most frequently handled by nurse telephone counselling, presented as frequency, incidence per 100,000 inhabitants/year and the proportion of total telephone contacts handled by nurse counselling per code/group

The ten RFE ICPC-2 codes most frequently handled by nurse telephone counselling within different age groups are presented in Table 4. A03 (Fever) and D01 (Abdominal pain) were among the top ten RFEs most often handled by nurse telephone advice in all six age groups. However, fever was significantly more prevalent in the youngest age group (0–5 years old), both in relation to other symptoms within this age group, and in relation to the prevalence of fever in the other age groups. It accounted for 21.6% of all advice given in the youngest age group, compared to 2.0–7.4% in the other age groups. H01 (Ear pain) was also a frequent symptom in the two youngest age groups, but was not among the symptoms most frequently handled by nurse telephone counselling in any of the other age groups. L17 (Foot/toe symptom) and N01 (Headache) were present in the three age groups from 6 to 59 years, while L02 (Back symptoms) was prevalent in all the age groups from 16 years and upwards. R02 (Shortness of breath/dyspnoea) was found among the most frequent RFEs only in the two oldest age groups.

**Table 4:** RFEs most frequently handled by nurse telephone counselling within different age groups

Age group	ICPC-2 code		Nurse telephone advice		
			Frequency	(%)	Incidence
0–5 years (*n* = 3364)	A03	Fever	727	(21.6)	333
	R05	Cough	176	(5.2)	81
	H01	Ear pain	156	(4.6)	71
	D10	Vomiting	117	(3.5)	54
	S06	Rash localized	85	(2.5)	39
	S07	Rash generalized	74	(2.2)	34
	D11	Diarrhoea	62	(1.8)	28
	D01	Abdominal pain	50	(1.5)	23
	R21	Throat symptom	50	(1.5)	23
	S12	Insect bite/sting	49	(1.5)	22
	ICPC-2 unknown		356		
6–15 years (*n* = 1435)	A03	Fever	106	(7.4)	49
	H01	Ear pain	68	(4.7)	31
	D01	Abdominal pain	63	(4.4)	29
	L17	Foot/toe symptom	47	(3.3)	22
	S13	Animal/human bite	43	(3.0)	20
	R21	Throat symptom	42	(2.9)	19
	R05	Cough	39	(2.7)	18
	S18	Laceration/cut	33	(2.3)	15
	N01	Headache	32	(2.2)	15
	S06	Rash localized	32	(2.2)	15
	ICPC-2 unknown		131		
16–29 years (*n* = 2904)	D01	Abdominal pain	150	(5.2)	69
	R21	Throat symptom	101	(3.5)	46
	A03	Fever	69	(2.4)	32
	A29	General symptom	68	(2.3)	31
	L02	Back symptom	63	(2.2)	29
	R05	Cough	62	(2.1)	28
	L17	Foot/toe symptom	53	(1.8)	24
	N01	Headache	51	(1.8)	23
	A97	Administrative contact	45	(1.5)	21
	S18	Laceration/cut	45	(1.5)	21
	ICPC-2 unknown		314		
30–59 years (*n* = 3528)	D01	Abdominal pain	130	(3.7)	60
	L02	Back symptom	99	(2.8)	45
	R05	Cough	92	(2.6)	42
	A29	General symptom	88	(2.5)	40
	L17	Foot/toe symptom	82	(2.3)	38
	A03	Fever	74	(2.1)	34
	A97	Administrative contact	72	(2.0)	33
	A13	Concern/fear medical treatment	67	(1.9)	31
	N01	Headache	65	(1.8)	30
	P02	Acute stress reaction	52	(1.5)	24
	ICPC-2 unknown		414		
60–79 years (*n* = 1255)	A13	Concern/fear medical treatment	40	(2.8)	18
	A29	General symptom	40	(2.8)	18
	R05	Cough	40	(2.8)	18
	D01	Abdominal pain	39	(2.8)	18
	L02	Back symptom	32	(2.3)	15
	R02	Shortness of breath/dyspnoea	31	(2.2)	14
	A97	Administrative contact	30	(2.1)	14
	A03	Fever	28	(2.0)	13
	P01	Feeling anxious/nervous/tense	26	(1.8)	12
	N17	Vertigo/dizziness	25	(1.8)	11
	ICPC-2 unknown		153		
80 + years (*n* = 915)	A13	Concern/fear medical treatment	41	(4.5)	19
	A28	Limited function/disability	36	(3.9)	16
	A96	Death	31	(3.4)	14
	R02	Shortness of breath/dyspnoea	31	(3.4)	14
	A97	Administrative contact	27	(3.0)	12
	A29	General symptom	26	(2.8)	12
	A03	Fever	20	(2.2)	9
	D01	Abdominal pain	20	(2.2)	9
	L02	Back symptom	18	(2.0)	8
	L14	Leg/thigh symptom	16	(1.7)	7
	ICPC-2 unknown		104		

The numbers are presented as frequency and incidence per 100,000 inhabitants/year

The five ICPC-2 codes most frequently handled by nurse telephone counselling in the age group 0–5 years old constituted about 40% of the total advice given in this group. Equivalent figures were 25% in the age group 6–15 years, and about 15% in the other age groups.

### Factors potentially influencing the decision to handle contacts solely by nurse telephone counselling

Of the contacts handled by nurse counselling, 34% were during daytime, 48% in the evening and 18% at night. Patients in the age groups 0–5 years and 30–59 years received 24 and 25% of all nurse advice respectively.

Table 5 presents the share of telephone contacts handled by nurse telephone counselling, stratified by age groups, genders and time of day. The results of the logistic regression analysis are displayed in Table 6. There were significant differences in the likelihood of handling telephone contacts by nurse counselling between the different times of the day, and between the different age groups (*P* < 0.001 for all variables).

**Table 5:** Proportion of nurse telephone counselling in different age groups and between the genders

Proportion of total telephone contacts handled by nurse telephone counselling									
				Distribution % (95% CIs)					
		Total % (95% CIs)		Daytime		Evening		Night	
All nurse telephone advice (*n* = 14,155)		23	(23–23)	21	(20–21)	23	(22–23)	31	(30–32)
Age group									
0–5	(*n* = 3364)	32	(31–33)	27	(26–28)	32	(31–32)	47	(46–49)
6–15	(*n* = 1435)	25	(24–26)	21	(20–22)	24	(23–25)	43	(40–45)
16–29	(*n* = 2904)	23	(22–24)	21	(20–22)	22	(22–23)	29	(28–30)
30–59	(*n* = 3528)	20	(19–21)	18	(18–19)	19	(19–19)	28	(27–29)
60–79	(*n* = 1408)	17	(16–18)	16	(15–16)	16	(16–17)	21	(20–22)
80≤	(*n* = 915)	17	(16–18)	16	(15–17)	17	(16–18)	22	(21–24)
Age unknown	(*n* = 601)	56	(56–58)	55	(53–57)	61	(59–64)	45	(41–50)
Sum	(*n* = 14,155)								
Gender									
Women	(*n* = 8163)	24	(24–24)	21	(21–21)	24	(23–24)	32	(32–33)
Men	(*n* = 5975)	22	(22–22)	20	(20–20)	22	(21–22)	29	(28–29)
Gender unknown	(*n* = 17)	16	(12–19)	20	(14–25)	11	(6–16)	16	(7–24)
Sum	(*n* = 14,155)								

The table presents the proportion of total telephone contacts handled by nurse counselling in different age groups and between the genders. Presented as total and for the different times of the day

**Table 6:** Likelihood (odds ratio) of nurse telephone counselling by time of day and age of patient

Variables	OR	95% CI	*P*-value
Time of day			
Daytime shift	Ref.		
Evening shift	1.11	1.10–1.16	<0.001
Night shift	1.81	1.71–1.92	<0.001
Age group			
0–5	Ref.		
6–15	0.71	0.66–0.77	<0.001
16–29	0.63	0.59–0.67	<0.001
30–59	0.53	0.50–0.56	<0.001
60–79	0.43	0.40–0.46	<0.001
80≤	0.45	0.41–0.49	<0.001

Logistic regression analyses using daytime shift and age group 0–5 years as reference

A larger share of the total telephone contacts was handled solely by nurses in the night shifts, compared with the evening and day shifts. Furthermore, the share of nurse telephone advice decreased as the patients’ age increased. In the age group 0–5 years, the total share of nurse telephone counselling was 32%, compared with 17% in the two oldest age groups. In the youngest age group, the share of total telephone contacts handled by nurse counselling increased from 27% in daytime, to 47% at night. In the two oldest age groups, equivalent numbers were 16% during the day and 21 and 22% during the night.

In the age group 80 years and above, 41% of the contacts were made by health personnel on behalf of the patient (the most frequent RFEs being A13 (Concern medical treatment), A28 (Limited function) and A96 (Death)), while this was the case in only 0.2% of the contacts in the youngest age group. There were no significant differences in the share of telephone contacts resolved by nurse advice between the weekdays Monday to Friday. However, there was a significant difference in the share of nurse advice during the week (mean nurse advice share: 25% (95% CIs 24–25)) compared to the weekend (mean nurse advice share: 20% (95% CIs 19–20)). In regard to the patients’ gender, only small variations were found; during the night 32% of all female contacts were resolved by nurse telephone advice compared with 29% of the male contacts.

## Discussion

The findings reveal that a large number of telephone contacts to the LEMCs were handled solely by nurse telephone counselling. The RFEs most frequently resolved by nurse telephone advice were fever, abdominal pain, cough, ear pain and general symptoms. Telephone contacts were more often resolved by nurse advice at night compared to day and evening, and more often during the week (Monday to Friday) compared to the weekends. A higher share of contacts with the youngest patients were handled by nurse telephone counselling compared with the oldest age group.

Of the total telephone contacts in our data material, 23% were handled solely by nurse counselling, which is equivalent to 65 nurse advice per 1000 inhabitants/year. This correlates with findings from previous years in the Watchtower project [2, 22]. It is also comparable with a study from the Netherlands where a nurse alone handled 27.5% of the telephone contacts [33]. Other studies from the Netherlands and UK have found that nurses handle as much as 40–50% of the contacts by nurse telephone advice [18, 23]. However, the literature seems somewhat divided on the effectiveness of nurse telephone triage. In one of the Dutch studies, it was indicated that high proportions of nurse telephone consultations were associated with increased probability of follow-up contacts, which in turn could lead to increased workload [18]. In contrast, studies from the UK found that nurse telephone consultation could reduce GP workload without an increase in adverse events [23]. Previous studies on the Norwegian OOH services conclude that two-thirds of the callers receiving nurse advice did not re-contact the OOH services, own GP or other sections of the healthcare services in the following week [19]. In the Norwegian OOH services, however, advising the patient to contact his or her own GP in practice hours is also an appropriate way of handling contacts that can wait. It is important to add that differences in the organization of the healthcare systems can make it difficult to directly compare study results between countries. In some European countries (e.g. UK and the Netherlands), and in the United States, self-referral to a hospital emergency department is possible. In Norway, a referral via the primary care sector is needed. Furthermore, in the Danish OOH service, it is mostly doctors who handle the phone calls, which is different from the other countries mentioned above, where nurses, physician assistants or secretaries handle the calls.

In our study, one fifth of all contacts handled by nurse telephone counselling were contacts regarding ICPC-2 chapter A (General and unspecified). The high frequency in this chapter can to some extent be explained by the code A03 (Fever). Fever was the symptom most often handled by nurse telephone counselling, and constituted 39% of all nurse advice given in chapter A. To the best of our knowledge, not many studies examine which RFEs telephone nurses resolve by advice. Findings from the United States also show that, among parents contacting a children’s careline regarding a child with fever, a majority followed nurse advice for home care, in spite of initially preferring to have their child seen by a medical doctor [25]. Furthermore, a Dutch study exploring determinants associated with nurse telephone advice alone presents similar findings to those found in our study [33]. A large share of contacts regarding earache, vomiting and fever were handled by nurse telephone consultations, consistent with our findings. Contacts regarding chest pain had the lowest share of nurse telephone advice, which is also similar to our study in which cardiovascular problems have the lowest share of contacts handled by nurse consultation. Furthermore, the Dutch study found that a greater share of telephone contacts was handled by nurse counselling at night, compared to day and evening. Contacts by the youngest age groups were more often handled by nurse advice compared to the older age groups. These findings are also in line with ours.

Several factors probably contribute to the large share of contacts handled in our study by nurse telephone counselling during the night, compared to day and evenings (Table 5); less resources at night-time, a desire not to wake the doctor on call, or the nurses may be stricter on what they would assign to be seen by a doctor during the night. Furthermore, our findings reveal that the major increase in nurse telephone advice during the night was due to changes in the two youngest age groups (Table 5). The lower share of nurse counselling during the weekend compared to the weekdays could be explained by the fact that it was not possible to advise the patients to contact their own GP the same day - or the day after, if a Saturday.

One reassuring finding is that the rate of nurse telephone advice declined as the patients’ age increased, indicating that nurses at the LEMCs make safe decisions. The oldest age group is a complex group, often presenting with several and more severe symptoms and comorbidities compared with younger patients. However, on our data form it is only possible to register one symptom per patient. Consequently, we do not know if the older patients in our group presented with a mix of symptoms. Another factor might be that contacts from the oldest age group were made much more frequently by health personnel on behalf of the patient. This means that the patient’s condition had often already been evaluated before contacting the OOH service. Furthermore, the youngest age group appeared to be more predictable regarding which RFEs the nurse resolved by nurse telephone advice. The five most commonly reported RFEs handled by nurse counselling in the youngest age group (fever, cough, ear pain, vomiting and localized rash), constituted around 40% of all advice given in this group. The type of contacts handled by nurse telephone counselling in the other age groups varied more.

Findings from Europe, the United States, New Zealand and Australia all show that children constitute a large group of the OOH contacts [2, 24, 27–29]. Studies from Denmark, Scotland and the Netherlands also report that the youngest age group receives telephone advice more frequently than the older age groups [29, 33, 37], and involves symptoms similar to those found in our study [33]. In a study from England reporting on young people’s (aged 0–15) use of the telephone service NHS Direct, it was found that 44 to 49% of all calls were resolved solely by nurse advice [28]. The high share of nurse telephone counselling in this group of patients might emphasize the parents’ need for advice and reassurance when their children are ill. Measures to meet this need could include public health centres to provide information and guidance to parents on the most usual symptoms and conditions that affect infants and young children.

### Strengths and limitations

This study adds important knowledge on the telephone nurses’ work in the Norwegian LEMCs. To our knowledge, this is the first study of its kind in Norway. One of the strengths of the study is the high numbers of observations. The data analysed were collected over a period of one year which minimises the influence of seasonal variations. Reports from previous years of the Watchtower project show that the quality of the data is good; in 2013 only 1% of the total records lacked at least one piece of information. Records lacking RFE ICPC-2 codes are not included in this number, as RFE is not marked as “compulsory” in the registering program.

In 2014, RFE ICPC-2 codes were missing in 11% of the total records, with a wide variation between individual OOH clinics, ranging from 2 to 21% [1]. These differences could reflect varying practices between the clinics in registration of the data. Of the contacts handled by nurse telephone counselling, 12% had no RFE ICPC-2 code registered. Even though the nurses have received standard training in how to register RFEs, there is still a potential for bias, for instance by recording a potential diagnosis instead of the symptom presented by the patient. However, symptoms are the dominating RFEs in our data material. The RFE mode of the ICPC system has also been found to be a reliable tool for registering RFEs in the primary care sector [38].

Although this study provides an important picture of the nurses’ contributions to the LEMCs, and which contacts are handled solely by nurse telephone counselling, it would have been of great interest to analyse associations between characteristics of the individual nurses and nurse telephone counselling strategies. However, such data were not available.

Comparing the Watchtower data with the yearly statistics on reimbursement claims in the Norwegian OOH service, it is evident that there is under-reporting of cases in the Watchtower database. Results from 2014 show a 15% deviation in the numbers of doctor consultations [1]. Under-reporting of nurse telephone consultations in our data material is probably of the same size, if not greater. However, it is not possible to calculate accurate figures. Reasons for missing records could be related to different priorities during busy times in the LEMCs.

When the Watchtower project was initiated in 2006, it was designed to be a representative sample of the OOH services in Norway [34]. However, changes in population and community structures might cause the representativeness to be less accurate in 2014 compared with 2006. In our study, we also excluded one of the Watchtowers due to organisational changes at the time of data collection.

Nonetheless, because of the high numbers of observations, we believe that our data still provide a reliable picture of contacts handled by nurse telephone counselling in the Norwegian OOH services.

## Conclusion

This study shows that nurses contribute significantly to patient management in the Norwegian OOH services. The findings can guide the process of developing training programmes for work in the LEMCs, especially in regard to the type of contacts the nurses have to manage and master. There is the potential for more nurse involvement in several of the RFEs which currently have a low share of nurse counselling. Finally, the study identifies areas that need further investigation. This includes the high share of nurse counselling provided during the night, compared to daytime, and the differences in the share of nurse advice between the youngest and the oldest age groups.

## Additional file

Additional file 1: Groups of related ICPC-2 RFE codes. A table showing groups of related ICPC-2 codes. (DOCX 13 kb)

## Abbreviations

GP: General practitioner
ICPC-2: International classification of primary care
LEMC: Local emergency medical communication centre
OOH: Out-of-hours
RFE: Reason for encounter
