# Supplementary material for: Telephone counselling by nurses in Norwegian primary care out-of-hours services: a cross-sectional study
Source: BMC Fam Pract. 2017 Sep 6;18:84. doi: 10.1186/s12875-017-0651-z (PMC5586064; doi:10.1186/s12875-017-0651-z)
Supplement: Additional file 1: — Groups of related ICPC-2 RFE codes. A table showing groups of related ICPC-2 codes. (DOCX 13 kb) [file 12875_2017_651_MOESM1_ESM.docx]

Additional file 1: Groups of related ICPC-2 RFE codes.

| **RFE Group** | **Included ICPC-2 codes** |
| --- | --- |
| **Abdominal pain** | D01, D02, D06, D88 |
| **Alcohol/substance abuse/addiction** | P15, P16, P17, -P18, P19 |
| **Anxiety** | P01, P02, P74, P75, P79 |
| **Chest symptom/condition** | A11, K01, K02, L04 |
| **Depression** | P03, P76, P77, P78 |
| **Diarrhoea/Vomiting** | D09, D10, D11, D70, D73 |
| **Ear symptom/condition** | H01, H70, H71 |
| **Eye symptom/condition** | F01, F02, F03, F15, F16, F70, F71, F72, F73, F76, F79, F85 |
| **Fears, concerns and worries** | All ‑-26 and ‑-27.  A13, A25, H15, K245, K254, W02, W21, X22, X23, X24, X25, Y24, Y25 |
| **General symptom** | All ‑29 |
| **Head/face symptom/condition** | N01, N03, N89, N90, N92, N95 |
| **Lower limbs symptom/injury/condition** | L13, L14, L15, L16, L17, L73, L75, L77, L78, L89, L90, L96 |
| **Mouth/teeth symptom/condition** | D19, D20, D82, D83 |
| **Neck/back symptom/condition** | L01, L02, L03, L84, L85, L86 |
| **Nose/sinus symptom/condition** | R07, R08, R09, R75 |
| **Respiratory infections** | R74, R77, R78, R81, R82, R83 |
| **Respiratory symptom/condition** | R01, R02, R03, R04, R05 |
| **Skin injury** | S15, S16, S17, S18, S19 |
| **Skin itching/rash** | S02, S06, S07 |
| **Throat symptom/condition** | R21, R23, R72, R76 |
| **Upper limb symptom/injury/condition** | L08, L09, L10, L11, L12, L72, L74, L92, L93 |
| **Urinary tract symptom/condition** | U01, U02, U06, U07, U71 |

Table first published in: Raknes G, Hunskaar S. Reasons for encounter by different levels of urgency in out-of-hours emergency primary health care in Norway: a cross sectional study. BMC Emerg Med. 2017;17:19 (licensed under CC BY 4.0).
